# Intravenous cyclophosphamide combined with steroids in pediatric onset severe lupus nephritis

**DOI:** 10.1007/s11255-012-0331-9

**Published:** 2012-12-07

**Authors:** Prayong Vachvanichsanong, Pornsak Dissaneewate, Edward McNeil

**Affiliations:** 1 Department of Pediatrics, Faculty of Medicine, Prince of Songkla University, Hat Yai, 90110 Songkla Thailand; 2 Epidemiology Unit, Faculty of Medicine, Prince of Songkla University, Hat Yai, 90110 Thailand

**Keywords:** Lupus nephritis, Systemic lupus erythematosus, SLE, Intravenous cyclophosphamide, IVCY

## Abstract

**Background:**

Intravenous cyclophosphamide (IVCY) has been used to treat severe lupus nephritis (LN) for many years. Because of the wide variety of manifestations of the condition and the long-term nature of the disease, outcomes vary widely.

**Objective:**

To evaluate and compare the immediate and long-term results of IVCY in pediatric onset severe LN and between patients with normal and abnormal initial renal function.

**Methods:**

Patients aged <18 years who attended the Department of Pediatrics, Prince of Songkla University, diagnosed with severe LN, and who were given a 36-month IVCY course, were included. Comparison of overall survival between the two groups was assessed using Kaplan–Meier survival curves.

**Results:**

108 patients with a mean age of 12.6 ± 2.7 years were studied, with a mean follow-up time of 5.7 ± 4.3 years. 48 patients completed the IVCY course. 36 patients had abnormal renal function and 72 patients had normal renal function at the start of therapy. Both groups responded well initially to treatment; proteinuria reduced to normal levels after 1 and 2 treatments in the normal and abnormal groups, respectively, while creatinine clearance returned to normal levels after 8 treatments in the abnormal group. Overall survival was not different between the two groups; however, the abnormal renal function group had a higher crude mortality rate than the normal group (13/36 vs 10/72, *p* value = 0.02). At the time of analysis, some patients who had completed their IVCY course still required other therapy to control their disease activity.

**Conclusion:**

Three years of IVCY treatment provided similar outcomes in both normal and abnormal renal function groups. Immediate outcomes were favorable but long-term remission was not promising.

## Background

Systemic lupus erythematosus (SLE) is a life-threatening illness which may lead to chronic disease affecting multiple organs. Induction of rapid and sustained remission is the optimal treatment result. In recent decades, multiple immunosuppressive drugs, such as cyclophosphamide, azathioprine, cyclosporine, or mycophenolate mofetil (MMF), have been trialed in combination with corticosteroids in an attempt to achieve long-term remission with minimal side effects [1–4]. Renal involvement is a major indicator of morbidity and mortality in SLE, and pediatric onset SLE is known to be more serious than in adult patients [5]. An ‘optimal therapy’ is difficult to determine, as the disease has a wide variety of manifestations [1, 6–8].

Currently, intravenous cyclophosphamide (IVCY) is the recommended treatment in severe lupus nephritis. Initial studies of IVCY therapy in severe lupus nephritis showed quite good outcomes in both adults and children; however, many subsequent studies with longer patient follow-up found that their results were not as good as the initial studies had promised with many patients relapsing and developing unfavorable, long-term side effects [9–13]. A preliminary report from our institution involving 16 children treated with IVCY showed satisfactory outcomes with minimal complications; however, the duration of remission was not evaluated due to the short follow-up period of that study [14].

The objectives of this study were to evaluate and compare the outcomes of IVCY treatment in childhood onset severe lupus nephritis in a large group of Thai children and between patients with normal and abnormal initial renal function.

## Methods

This retrospective study examined the medical records of patients diagnosed with SLE based on the American Rheumatism Association 1982 revised criteria [15], with LN WHO class IV or who had severe glomerulonephritis, defined by rising serum creatinine or heavy proteinuria, who had not responded to corticosteroid therapy or corticosteroids combined with oral cyclophosphamide. All patients who attended the Department of Pediatrics, Prince of Songkla University Hospital between October 1993 and December 2008 were included. During this period in our institution, patients who had LN WHO class IV or severe glomerulonephritis were offered a 36-month course of IVCY therapy, totaling 17 treatments, monthly for 7 treatments followed by every 3 months for 10 treatments. One treatment consisted of cyclophosphamide in 100 ml of 5 % dextrose in water, administered intravenously for 1 h at 500, 750, and 1,000 mg/body surface area (BSA), on the first, second, and thereafter treatments, respectively. At each treatment, a half isotonic saline solution was infused for 24 h following the treatment at 2,000 ml/m^2^/day to ensure the patient did not become dehydrated and to avoid hemorrhagic cystitis. 2-mercaptoethane sulfonate sodium (MESNA) was not used in any patient.

Oral prednisolone and pulse methylprednisolone were combined, adjusted to the clinical and serological status of each individual patient, and antihypertensive drugs and diuretics were given as required. Treatment was temporarily suspended if the patient developed infection, fever, or if the white blood cell count (WBC) dropped below 3,000/mm^3^. Anti-emetic drugs were prescribed to patients who experienced nausea and vomiting.

Each patient’s weight, height, BSA, blood pressure, urinalysis, complete blood count (CBC), 24-h protein and creatinine, blood urea nitrogen, serum creatinine, antinuclear antibodies (ANA), anti-double-strand-DNA (anti-ds-DNA), and complement component 3 (C_3_) were measured before every treatment. 24-h urine protein and creatinine levels were regarded as unreliable and excluded from analysis if urine creatinine was less than 10 mg/kg/day or greater than 30 mg/kg/day. Creatinine clearance (CCr) was calculated by a standard formula using 24-h urine collection (excluded as above if CCr greater than 250 ml/min/1.73 m^2^).

The patients were divided into two groups, based on the initial serum creatinine and creatinine clearance levels—the first group being those with creatinine clearance less than 90 ml/min/1.73 m^2^ or serum creatinine level higher than the upper limit of the normal range for age and sex (abnormal renal function group), and the second group being the remaining patients (normal renal function group), and the results of treatment of the patients in the two groups were compared at the end of the treatment course. The status of medication, such as prednisolone or other immunosuppressive drugs, at the time of analysis was used to evaluate long-term treatment outcomes. Chronic kidney disease (CKD) was defined if a patient had abnormal renal function lasting for more than 6 months.

Statistical analysis was performed using R software, version 2.10.1 [16]. Pearson’s chi-squared test or Fisher’s test as appropriate were used for testing associations between two or more variables. Kaplan–Meier survival curves were used to estimate and compare survival rates, and the log-rank test was used to compare survival curves between the two groups. *p* values less than 0.05 were considered significant.

This study was conducted with the approval of the Ethics Committee of Prince of Songkla University.

## Results

During the study period, a total of 108 (19 male, 89 female) patients with a mean age at diagnosis of 11.4 ± 2.6 years (range 5.4–16.9) and age at start of IVCY therapy 12.6 ± 2.7 years (range 5.5–20.6) received corticosteroid treatment combined with IVCY. The mean follow-up time was 5.7 ± 4.3 years (median 3.6 years, range 30 days–14.7 years). 92 patients had LN class IV prior to starting treatment, 8 patients had LN class II but impaired renal function, 2 patients had LN class V with renal failure, 1 patient had LN class III, and 5 patients had not had a biopsy.

There were 72 patients who had normal renal function and 36 who had abnormal renal function (34 with creatinine clearance <90 ml/min/1.73 m^2^, 2 with abnormally high creatinine level for age and sex). Table 1 shows a comparison of patient characteristics, baseline laboratory findings, LN classification, and follow-up status at the end of the study between those with initial abnormal renal function and those with normal renal function. There were no significant differences between the two groups in terms of sex, age, and LN class; however, a higher proportion of deaths occurred in the abnormal renal function group. Baseline urinary protein levels were significantly higher in the abnormal renal function group, while hemoglobin and white blood cell levels were significantly lower.

**Table 1:** Comparison of patient characteristics, baseline laboratory levels, initial LN classification, and follow-up status by initial renal function group

	Initial renal function		Total	*p* value
	Normal	Abnormal		
Sex, *N* (%)				0.66
Male	14 (19.4)	5 (13.9)	19 (17.6)	
Female	58 (80.6)	31 (86.1)	89 (82.4)	
Age (years), mean (SD)	12.2 (2.5)	13.3 (2.9)		0.05
Baseline laboratory levels, median (IQR)				
Urinary protein (mg/kg/day)	1,320 (355–2,257.5)	2,200 (1,090–3,670)		0.03
Creatinine clearance (mL/min/1.73 m^2^)	102.7 (93.9–123.2)	64.8 (57.6–83.2)		<0.001
Creatinine (mg/dL)	0.7 (0.6–0.8)	0.9 (0.8–1.3)		<0.001
Hemoglobin (g/dL)	10.9 (9.8–11.7)	9.1 (7.9–10.1)		<0.001
WBC (cells/mm^3^)	9,400 (6,700–11,950)	6,700 (4,850–11,500)		0.046
Platelets (cells/mm^3^)	301,500 (240,750–373,000)	320,000 (160,000–364,000)		0.23
Antinuclear antibody (titer)	160 (35–640)	160 (35–640)		0.93
C_3_ (mg/dL)	52 (33.5–78.5)	40 (21–62.5)		0.86
LN Class, *N* (%)				0.16
II	4 (5.6)	5 (13.9)	9 (8.3)	
III	0 (0)	1 (2.8)	1 (0.9)	
IV	63 (87.5)	28 (77.8)	91 (84.3)	
V	2 (2.8)	0 (0)	2 (1.9)	
No biopsy	3 (4.2)	2 (5.6)	5 (4.6)	
Completed IVCY course, *N* (%)				0.15
No	44 (61.1)	16 (44.4)	60 (55.6)	
Yes	28 (38.9)	20 (55.6)	48 (44.4)	
Follow-up time (years), median (IQR)	3.18 (1.47–5.46)	5.09 (3.58–6.77)		0.006
Status, *N* (%)				0.05
Alive	46 (63.9)	15 (41.7)	61 (56.5)	
Lost to follow-up	7 (9.7)	3 (8.3)	10 (9.3)	
Dead	10 (13.9)	13 (36.1)	23 (21.3)	
Referred	9 (12.5)	5 (13.9)	14 (13.0)	
Total	72	36	108	

**Fig. 1:**
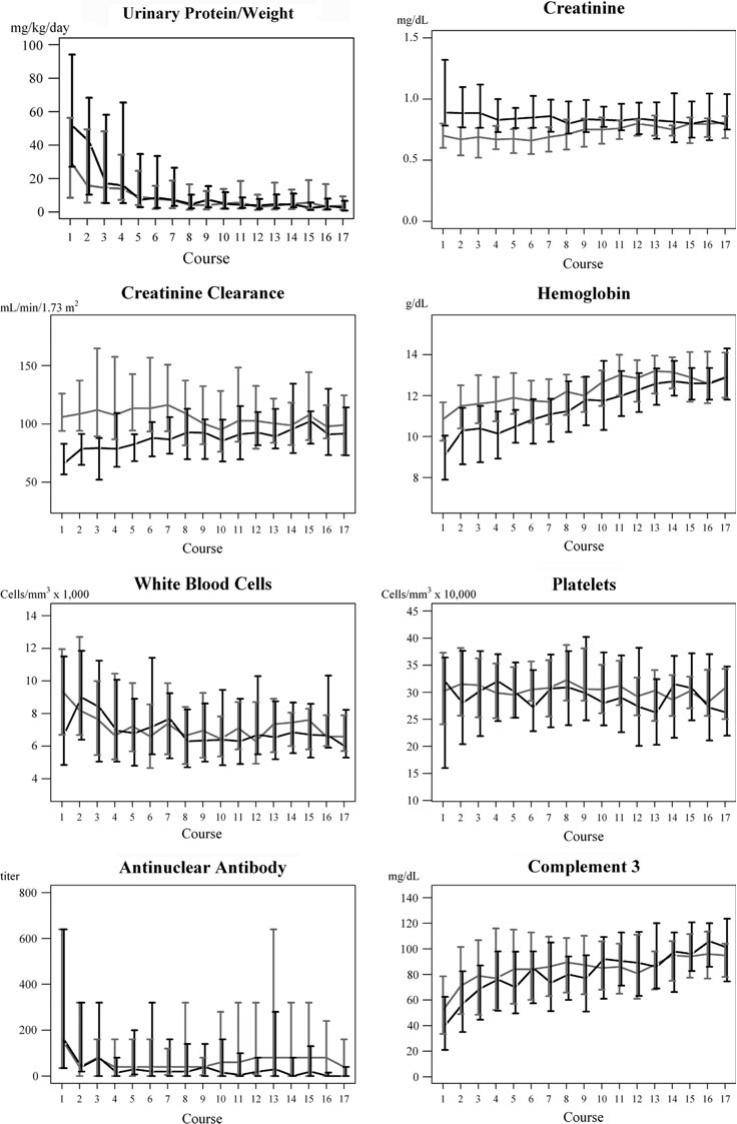
Comparison of serum creatinine, creatinine clearance, urinary protein, hemoglobin, white blood cell count, platelet count, C_3_, and antinuclear antibody over the 17-treatment course of IVCY between patients with abnormal renal function (*black*) and normal renal function (*gray*) (*N* = 36 vs 72)

Figure 1 shows the comparison of laboratory findings of the two groups of patients during IVCY therapy. Serum creatinine and creatinine clearance were not changed during the period of therapy for patients with normal renal function. For patients with abnormal renal function, the creatinine level and creatinine clearance gradually improved and reached normal levels after the 8th treatment; however, only 20 of these patients completed the full course of 17 treatments. Proteinuria dramatically decreased after 1 treatment of IVCY in patients who had normal renal function, while urinary protein decreased after 2 treatments of IVCY in patients who had abnormal renal function.

Hemoglobin levels increased steadily in both groups, more rapidly for the patients with abnormal renal function; however, levels were lower initially in this group. White blood cells were suppressed by therapy in both groups. Platelets were steady and within normal ranges among patients in the normal renal function group; however, no patient had a platelet count less than 100,000 cells/mm^3^.

In all cases, the ANA titer decreased immediately after the first treatment and remained low until the end of the course. C_3_ increased to normal levels after the 14th treatment in both groups.

Thirty-two patients developed acute kidney injury (AKI), of whom 7 died, 18 resolved, and 7 progressed to CKD. Two patients also had CKD at the time they were first seen by us, but both died later after initiation of the IVCY treatment.

Twenty-five patients had a repeat renal biopsy performed after the end of their course of IVCY (within 100 days), of whom 10 LN class IV remained class IV, 13 LN class IV patients improved, one patient with LN class IV progressed to end stage renal disease (ESRD), and one LN class II remained LN class II.

The major complications of therapy were infections and hemorrhagic cystitis. Secondary malignancy was not found in any patients. Nausea and vomiting were the most common side effects. Three patients who had straight hair prior to IVCY treatment grew curly hair after alopecia.

As shown in Table 1, at the end of the study, twenty-three patients had died, 14 were referred to other clinics, 10 patients were lost to follow-up, and 61 were still being followed. Forty-eight patients had completed the full 17-treatment course of IVCY therapy, and of the 26 who were also still being followed up, 9 (19 %) were free of medication, 12 were on prednisolone alone, while 5 were on prednisolone plus one other immunosuppressive drug (1 azathioprine, 4 MMF). One patient who was on only prednisolone at the end of the study had been treated with azathioprine and MMF after completing the full IVCY course but the proteinuria had persisted, while one patient who had not received treatment for inactive SLE developed ESRD and was subsequently on dialysis.

At the time of analysis, 31 patients were still maintaining their treatment program, while 29 patients had begun but not completed the full course: 15 patients had died, 2 had been referred to other clinics, 8 had been lost to follow-up, and 4 patients had had their therapy terminated (two for severe vomiting, and one each for hemorrhagic cystitis and ESRD).

Of the 48 cases who completed therapy, 30 patients relapsed; 16 in the normal renal function group and 14 in the abnormal renal function group. The most common site of relapse was renal related.

**Fig. 2:**
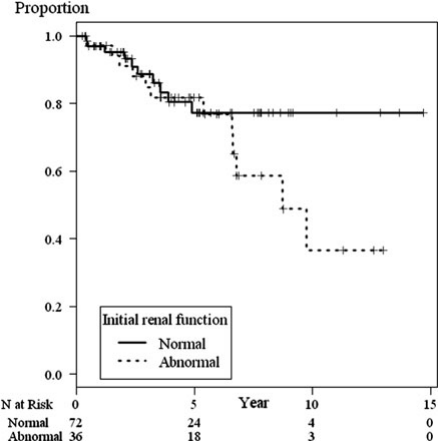
Survival curve of SLE patients treated with IVCY by initial renal function group

Figure 2 shows the patient survival curve of SLE patients separated by initial renal function group from the start of treatment with IVCY. There was no significant difference in overall survival between the two groups, although the crude mortality rate was higher in the group of patients who had abnormal renal function at the start of treatment (10/72 vs 13/36, *p* value = 0.02). The overall survival rates at 2 and 5 years were 93 % (*n* = 77, 95 % CI: 87–98 %) and 78 % (*n* = 42, 95 % CI: 70–88 %), respectively. The median survival time of the normal renal function group was undefined. For the abnormal renal function group, the median survival time was 8.7 years (*p* = 0.15).

## Discussion

Three years of therapy with IVCY is a long-term treatment. In addition, SLE is not a common disease in children, so a follow-up period of many years is required in order to properly evaluate long-term outcomes. Our study on the efficacy of IVCY in pediatric onset SLE in a single center included both a large number of patients and a long duration of follow-up. We found that IVCY therapy during the acute phase provided quite a good response in terms of clinical activity of SLE, serum creatinine, creatinine clearance, proteinuria, and serological activity of the disease. The maintenance phase is as important as the treatment stage, as the minimum dosage needs to be the best achievable compromise between minimal adverse reaction and optimal therapy.

Patients with abnormal renal function also achieved a good initial response in terms of reduced proteinuria, although these patients had a higher baseline level, and it took longer for them to return to normal levels than for patients with normal renal function. Creatinine clearance took longer to return to normal levels than proteinuria in patients with abnormal renal function.

Our study showed that IVCY can induce remission of SLE, although it is rarely long-term in the context of such a chronic disease, with the disease reawakening in many cases. A similar study in adults [17], and one by Al Salloum et al. [18] in 9 children (4 LN III, 5 LN IV), showed improvement of renal function during treatment but the improvement was not sustained—5 patients developed CKD within 2 years after completing the 3-year IVCY course (follow-up time >5 years from the beginning of IVCY treatment).

An earlier study we conducted involving 21 of our patients, of which 16 completed the 17-treatment course over three years and were then followed up for a median of 6.3 years, showed that after a few months of therapy, hemoglobin, proteinuria, and serology tests dramatically improved but serum creatinine and creatinine clearance did not [14].

Lehman and Onel [19] reported that in 16 children with lupus nephritis treated with 3 years of IVCY, the renal biopsy activity index significantly improved (*p* < 0.001), the mean 24-h urine protein significantly decreased (*p* < 0.05), and the mean creatinine clearance significantly increased (*p* < 0.01). The results of our treatments were not as good in terms of improved LN classifications. In the Lehman and Onel study, all 11 LN class IV cases changed, with most improving, 3 to LN V, 1 to LN III, 6 to LN II, and 1 to LN I [19]. Our study had 25 repeat renal biopsies after the complete 17-treatment course of IVCY, but only 13 patients with LN class IV changed to other classes (11 had no change and one progressed to ESRD).

In our previous study, LN class IV patients had a similar outcome compared to LN class II patients, indicating that our treatment regimen for LN class IV patients was effective, as in general LN class IV patients have a poorer prognosis than LN class II patients [20].

Infection is a common complication in SLE patients treated with IVCY therapy, as we previously reported, with opportunistic infections being the most common [21]. Severe infections were found in 36.9 % (31/84) of those cases, of whom 13 died (41.9 %). Fungal and Gram negative bacteria were the most common organisms responsible for severe infection. Pulse methylprednisolone usage, fungal infection, and renal failure were significant risk factors for mortality [21].

Although there was no difference in survival rates between the two groups, as assessed by the log-rank test, the crude mortality rate in the impaired renal function group was significantly higher. A likely reason for this is that these patients would have progressed to end stage renal failure, and in our institution, long-term dialysis and renal transplantation are not routinely performed. Chronic dialysis and renal transplantation for children in Thailand are still limited for economic reasons, which inevitably make the mortality rate higher than in countries with more resources.

Although cyclophosphamide has been reported to cause secondary malignancy, it is difficult to differentiate this from other possible causes. Malignancy was not identified in any patients in our study; however, the follow-up time was probably too short for malignancy to develop.

Ovarian dysfunction secondary to IVCY therapy could not be assessed since our patients were mostly teenagers, and although irregularity of menstruation was noted, this is not uncommon in this age group so any possible relationship with the treatment could not be assessed. In our study, out of 26 patients who completed the 17-treatment course and were alive at the time of analysis, 24 were girls with a median age of 21 years. Two were aged less than 15 years. At least three patients out of 22 potentially fertile women became pregnant after therapy; thus, it is clear that IVCY therapy does not guarantee ovarian dysfunction in this age group. A study in Chinese adults showed that older age and higher accumulative doses of cyclophosphamide were independent risk factors of ovarian failure [22]. Another study in 19 females with an average age of 24 years found that 3 patients had ovarian failure, while 3 achieved full-term pregnancy [23]. Other side effects were also not assessed in this study due to the small sample size. Studies with larger patient numbers and long-term follow-up are required to fully investigate this issue.

In our preliminary study of 20 patients, patient survival at 5 years was 87 % (95 % CI 59–97) [14]. In this larger and longer study, the rate was only slightly smaller (78 %, 95 % CI 70–88). However, such good results depend on completing the lengthy 36-month IVCY therapy, and this is not easily achieved for various reasons. Additionally, some patients die during treatment, and many drop out either due to being referred to other clinics or lost to follow-up. In our study, less than 50 % of those who started treatment could be evaluated at 5 years.

This retrospective study on a group of Thai children has some limitations. First, the severity of LN varied widely, and thus, other therapies were added to IVYC according to the disease exacerbation. Second, many children were referred elsewhere, such as other clinics, after completion of treatment, due to entering adulthood or for other reasons. Third, there may have been other factors that were not collected or measured which could not be controlled for in the analysis. Finally, the follow-up rate after 5 years was poor due to families moving to other areas of the country.

## Conclusion

IVCY combined with steroid therapy in Thai pediatric onset severe lupus nephritis patients, initially with both impaired and normal renal function, achieved a favorable outcome in terms of induction of disease remission and stability of disease during maintenance therapy. However, the long-term outcome after therapy is still less than satisfactory; although some patients remained medication free for many years of follow-up at the end of study, most required a return to medication to control their disease activity.
